# Supplementary material for: Optimizing the sensitivity of detection of respiratory syncytial virus infections in longitudinal studies using the combination of weekly sample testing and biannual serology
Source: medRxiv. 2025 Sep 19:2025.09.18.25336083. Preprint. [Version 1] doi: 10.1101/2025.09.18.25336083 (PMC12458533; doi:10.1101/2025.09.18.25336083)
Supplement: Supplement 1 [file NIHPP2025.09.18.25336083v1-supplement-1.pdf]

medRxiv preprint doi: https://doi.org/10.1101/2025.09.18.25336083; this version posted September 19, 2025. The copyright holder for this preprint (which was not certified by peer review) is the author/funder, who has granted medRxiv a license to display the preprint in perpetuity.

It is made available under a CC-BY 4.0 International license .

Supplemental table 1: comparison of demographics by subset inclusion criteria

	Overall N = 245[1]	Excluded from analysis N = 51[1]	<70% weekly samples, but serum ≥18M N = 93[1]	70% ≤ weekly samples <90% N = 48[1]	≥90% weekly samples N = 53[1]	p-value[2]
**Nasal swab adherence**	61 (22, 88)	29 (13, 54)	29 (11, 51)	80 (74, 87)	94 (91, 95)	<0.001
**Maternal age**	29.6 (25.7, 33.2)	26.1 (23.2, 30.3)	28.7 (25.2, 31.3)	31.6 (28.3, 35.0)	33.0 (30.0, 34.6)	<0.001
**Race**						<0.001
Black	107 (44%)	25 (49%)	65 (70%)	11 (23%)	6 (11%)	
White/Other	138 (56%)	26 (51%)	28 (30%)	37 (77%)	47 (89%)	
**Hispanic Ethnicity**	6 (2.4%)	0 (0%)	1 (1.1%)	2 (4.2%)	3 (6%)	0.2
**Insurance type**						<0.001
Private	106 (43%)	13 (25%)	15 (16%)	30 (63%)	48 (91%)	
Public	139 (57%)	38 (75%)	78 (84%)	18 (38%)	5 (9.4%)	
**Marital status**						<0.001
Married/Parter	163 (67%)	29 (57%)	47 (51%)	40 (83%)	47 (89%)	
Single	82 (33%)	22 (43%)	46 (49%)	8 (17%)	6 (11%)	
**Maternal Education**						<0.001
<=HS	115 (47%)	34 (67%)	63 (68%)	13 (27%)	5 (9.4%)	
>HS	130 (53%)	17 (33%)	30 (32%)	35 (73%)	48 (91%)	
**Family Income**						<0.001
<$25k	88 (36%)	27 (53%)	49 (53%)	10 (21%)	2 (3.8%)	
$25k-$50k	49 (20%)	13 (25%)	26 (28%)	7 (15%)	3 (5.7%)	
>$50k	108 (44%)	11 (22%)	18 (19%)	31 (65%)	48 (31%)	
**Infant sex**						0.2
Female	127 (52%)	31 (61%)	50 (54%)	24 (50%)	22 (42%)	
Male	118 (48%)	20 (39%)	43 (46%)	24 (50%)	31 (58%)	
**Delivery mode**						0.14
C Section	94 (38%)	16 (31%)	30 (32%)	23 (48%)	25 (47%)	
Vaginal	151 (62%)	35 (69%)	63 (68%)	25 (52%)	28 (53%)	
**Birthweight (kg)**	3.29 (2.95, 3.53)	3.27 (2.87, 3.46)	3.22 (2.83, 3.50)	3.32 (3.09, 3.62)	3.37 (3.07, 3.61)	0.2
**Breastfeeding initiation**						0.023
Did not initiate BF	33 (13%)	10 (20%)	18 (19%)	3 (6.3%)	2 (4%)	
Initiated BF	212 (87%)	41 (80%	75 (81%)	45 (94%)	51 (96%)	
**Days of BF**	83 (16, 312)	46 (9, 138)	33 (2, 123)	227 (47, 430)	310 (122, 428)	<0.001

[1]Median (Q1, Q3); n (%)

[2]Kruskal-Wallis rank sum test; Pearson's Chi-squared test; Fisher's exact test

Supplemental Table 2: Distribution of new serologically-identified RSV infections in 194 PREVAIL children

		**All new concentration change-identified infections** ***N*=133**	**Method used to identify infection**		
			**IgA only** ***n*=51 (38%)**	**IgG only** ***n*=3 (2%)**	**Both** ***n*=79 (60%)**
**Age category**	< 6 months	2 (2%)	2 (4%)	0 (0%)	0 (0%)
	6-11 months	11 (8%)	9 (18%)	0 (0%)	2 (3%)
	12-17 months	59 (44%)	19 (37%)	2 (67%)	38 (48%)
	18-24 months	61 (46%)	21 (41%)	1 (33%)	39 (49%)
**Infection number**	First	57 (43%)	21 (41%)	0 (0%)	36 (46%)
	Second	61 (46%)	28 (55%)	2 (66%)	31 (39%)
	Third	15 (11%)	2 (4%)	1 (33%)	12 (15%)

Legend:

Mixed effects classification and regression tree (CART) analysis was used to identify thresholds of positivity using pre-fusion F IgA and IgG assays from serum collected at 6 weeks and 6, 12, 18, and 24 months of age. CART-derived thresholds of >2.02 change in $\log_{10}$ concentration of IgA or a >0.32 change in $\log_{10}$ concentration of IgG were applied to PREVAIL participants who participated in the study at least 18 months and were either ≥70% adherent in weekly sample submission or provided a serum sample at 18 or 24 months of age.

Supplemental table 3: Comparison between concentration change method and ROC-derived seropositivity in 194 evaluable children in the PREVAIL Cohort

		Concentration change<br>log_{10} AU<br>IgA >0.202 or IgG>0.32		
		Pos	Not Pos	% agreement
ROC-derived IgA seropositivity	Pos	140	0	98.5%
	Not pos	3*	51	

Legend:

Mixed effects classification and regression tree (CART) analysis was used to identify thresholds of positivity of >2.02 change in log_{10} concentration of IgA or a >0.32 change in log_{10} concentration of IgG as the most predictive threshold for use in identifying RSV infections. Seropositivity by the laboratory-determined ROC curves was limited to IgA, as 100% of children were seropositive by IgG from birth until age 6 months

- The three participants identified as seropositive using the CART-defined thresholds were positive by IgG only and confirmed as positive by IgG in the ROC analysis.
